# Ophthalmologic assessment and intracranial pressure in children: diagnostic methods, clinical correlations, and future directions

**DOI:** 10.3389/fped.2026.1792500

**Published:** 2026-05-07

**Authors:** Elena Hernández-García, Barbara Burgos-Blasco, Noemi Güemes-Villahoz, Laura Morales-Fernandez, Jose Ignacio Fernandez-Vigo, Enrique Santos-Bueso, Rosario Gomez-de-Liaño, Julian García-Feijóo

**Affiliations:** 1 Servicio Oftalmologia, Hospital Clínico San Carlos, Instituto de Investigación Sanitaria del Hospital Clínico San Carlos (IdISSC), Madrid, Spain; 2 Departamento de Oftalmología, Inmunología y ORL, Facultad de Medicina, Universidad Complutense de Madrid, Madrid, Spain

**Keywords:** children, intracranial hypertension, intracranial pressure, papilledema, pediatric

## Abstract

Increased intracranial pressure (ICP) in the pediatric population represents a critical and potentially life-threatening condition that may lead to severe neurological sequelae, including brain herniation, cerebral ischemia, and irreversible neurological impairment, such as visual loss. The diagnosis of elevated ICP in children is particularly complex, as clinical manifestations are frequently subtle and non-specific, especially in infants and young children. Symptoms such as irritability, poor feeding, lethargy, and failure to thrive often overlap with a wide range of common pediatric disorders, thereby contributing to delayed diagnosis and an increased risk of adverse neurological outcomes. Within this context, ophthalmologic evaluation constitutes a key non-invasive tool in the early detection of increased ICP. Fundoscopic examination provides critical diagnostic information, with optic disc abnormalities among the most sensitive markers of elevated ICP. However, these findings may be less pronounced or exhibit distinct characteristics in children compared to adults, and their interpretation is further complicated by age-related variability in ocular anatomy and intracranial pressure norms. This review provides an integrated synthesis of current diagnostic strategies for assessing elevated ICP in children, with a particular focus on ophthalmologic assessment. It examines the association between specific ocular findings and ICP, with the aim of improving early recognition, guiding clinical management, and enhancing patient outcomes. Furthermore, it addresses the inherent challenges of pediatric ICP diagnosis and highlights key gaps in the literature that warrant further investigation.

## 1 Introduction

Increased intracranial pressure (ICP) in children is a critical medical condition characterized by abnormally elevated pressure within the cranial cavity, which may result in severe neurological complications, including brain herniation, ischemia, and permanent neurological damage, including vision loss. It constitutes a potentially life-threatening emergency that requires timely recognition and prompt management. Because the clinical signs and symptoms of increased ICP in children can be subtle or nonspecific, diagnosis may be delayed, thereby increasing the risk of irreversible neurological injury (1, 2).

In the pediatric population, the diagnosis of increased ICP presents unique challenges. One of the most significant obstacles is the atypical clinical presentation of elevated ICP in children. Unlike adults, who may present with classic symptoms like headache, nausea, vomiting, or altered mental status, children, especially infants and toddlers, may show non-specific symptoms such as irritability, poor feeding, lethargy, and failure to thrive. These symptoms are often non-specific and overlap with other common pediatric conditions, which can delay an accurate diagnosis. Furthermore, the neurological examination in younger children is inherently more difficult, as it relies heavily on the child's ability to cooperate with the clinician. This can make the detection of focal neurological deficits or subtle signs of increased ICP more challenging (3, 4).

Cerebrospinal fluid (CSF) opening pressure measured during lumbar puncture remains a key reference standard in the evaluation of suspected increased ICP. When performed under appropriate conditions, it provides a direct estimation of ICP and can support or confirm the diagnosis. However, its interpretation in the pediatric population requires caution, as normal values vary with age, patient positioning, level of sedation, and clinical context. Moreover, lumbar puncture is not always feasible or safe, particularly in cases where significantly elevated ICP is suspected due to the risk of brain herniation. Therefore, although CSF opening pressure is an essential diagnostic parameter, it must be integrated with clinical findings and non-invasive assessments.

Among the many diagnostic tools available, ophthalmologic examination plays a central role in identifying elevated ICP, serving as a non-invasive and highly informative component of the diagnostic process. The ophthalmologic examination in children with suspected increased ICP is of paramount importance, as changes in the fundus are among the most sensitive indicators of increased ICP. When detected early, these findings may serve as an early warning sign for potential neurological emergencies, prompting further diagnostic investigation and intervention. They are also valuable in assessing the severity and progression of elevated ICP. However, it is essential to note that the ophthalmologic signs of increased ICP in children can often be subtle, especially in younger patients, or may present differently than in adults (4–6).

Another key factor to consider is the variability of normal values in pediatric ophthalmology. The normal appearance of the optic disc, the intraocular pressure (IOP), and even the size and shape of the optic nerve head can differ significantly with age, which complicates the interpretation of ophthalmologic findings. Moreover, normal ICP levels vary depending on a child's age and developmental stage, making it crucial to assess ophthalmologic findings in the context of the child's growth and neurological development. Hence, the challenge of interpreting the results of an eye exam in a pediatric population requires an experienced ophthalmologist (7, 8).

Despite the recognized importance of ophthalmologic findings in the evaluation of increased ICP, the existing literature remains fragmented. Previous studies have often focused either on neurological assessment or on isolated ophthalmologic features, with limited integration of these perspectives, particularly in the pediatric population. This review aims to address this gap by providing an integrated and up-to-date synthesis of diagnostic methods for assessing elevated ICP in children, with a particular emphasis on ophthalmologic evaluation. Specifically, it explores the relationship between ophthalmologic findings and ICP, highlights age-related differences in presentation, and discusses the strengths and limitations of current diagnostic approaches. By doing so, this review offers a clinically oriented framework to support early diagnosis and improve patient outcomes, while also identifying key areas where further research is needed.

## 2 Methodology

This study is a narrative review aimed at providing a comprehensive overview of the role of ophthalmologic assessment in the evaluation of increased ICP in children. A literature search was conducted in major electronic databases, including PubMed/MEDLINE, Scopus, and Web of Science, covering studies published up to 2025. The search strategy combined relevant keywords such as “intracranial pressure,” “pediatric,” “children,” “papilledema,” “optic nerve,” “ophthalmologic examination,” and “fundoscopy”. Articles were selected based on their relevance to the topic, with particular emphasis on studies addressing the relationship between ophthalmologic findings and ICP in pediatric populations. Both original research articles and relevant reviews were considered. Exclusion criteria included studies not involving pediatric patients, articles not available in English, and studies lacking clinical relevance to the objectives of this review. Given the narrative nature of this review, a formal assessment of study quality or risk of bias was not systematically performed. However, priority was given to high-quality and widely cited studies.

## 3 Pathophysiological mechanisms that explain the relationship between ocular variables and intracranial pressure

The anatomical and physiological continuity between the optic nerve and the central nervous system provides a robust framework for understanding how ICP influences ocular structures. The optic nerve, an embryological extension of the diencephalon, is enclosed by the dura mater, arachnoid, and pia mater, forming a tubular meningeal sheath that extends the intracranial subarachnoid space (SAS) into the orbit. This perineural compartment contains CSF and communicates with the intracranial SAS, allowing variations in ICP to be transmitted directly along the optic nerve, although local anatomical factors may influence CSF dynamics (9, 10). Accordingly, the cranial SAS is continuous with the optic nerve SAS (ONSAS), which is a multichambered tubular structure that terminates in a blind end posterior to the globe (11, 12). Within the optic nerve, CSF extends from the chiasmatic cistern through the canalicular segment into the intraorbital portion of the nerve, which ends in a blind pouch posterior to the globe (13). This configuration may predispose the optic nerve to alterations in pressure transmission and CSF flow under conditions of raised ICP (14–16).

The ONSAS terminates abruptly at the lamina cribrosa, a critical biomechanical interface that separates the IOP anteriorly from the retrolaminar CSF pressure posteriorly (10, 17). This anatomical arrangement establishes a translaminar pressure gradient that is dynamically influenced by fluctuations in IOP and ICP. As a result, the intraocular portion of the optic nerve is simultaneously exposed to two opposing pressure environments: the IOP within the globe and the CSF pressure in the ONSAS, separated structurally by the lamina cribrosa and the peripapillary scleral flange (17).

The translaminar pressure difference (TLPD), commonly defined as the difference between IOP and ICP, quantifies the net pressure acting across this interface (18, 19). Disruptions in TLPD, whether due to elevated IOP or reduced ICP, have been associated with deformation of the lamina cribrosa, impaired axonal transport, reduced retrolaminar perfusion, and increased susceptibility to glaucomatous optic neuropathy (17, 19).

Papilledema represents the most widely recognized ocular manifestation of elevated ICP and reflects the vulnerability of retinal ganglion cell axons as they traverse the lamina cribrosa. Increased CSF pressure within the ONSAS raises retrolaminar tissue pressure and compresses the prelaminar and laminar segments of the optic nerve. When retrolaminar pressure exceeds IOP, the translaminar gradient reverses, disrupting both orthograde and retrograde axoplasmic transport (16, 20). Obstruction of axoplasmic flow leads to intracellular accumulation of organelles and cytoskeletal material proximal to the lamina cribrosa, resulting in optic disc edema (21). Clinically, this manifests as optic disc elevation, blurred margins, vascular obscuration, and, in more advanced cases, peripapillary hemorrhages (15, 22). Structural changes typically precede functional deficits, and children may present with marked papilledema while maintaining relatively preserved visual acuity and visual fields.

Ongoing research aims to elucidate the mechanisms by which increased ICP leads to papilledema. Two principal theories predominate: the mechanical theory and the ischemic theory. The *mechanical theory* proposes that elevated ICP causes direct compression of prelaminar optic nerve axons, resulting in impaired axoplasmic transport and accumulation of axoplasm at the level of the scleral lamina (23). Supporting this hypothesis, Howden et al. (24) demonstrated that CSF velocity decreases with increasing distance from CSF inlets, suggesting that any reduction in CSF flow would preferentially affect distal CSF compartments, including the SAS of the optic nerve, which is anatomically distant from the ventricles and does not permit continuous flow.

The *ischemic theory* posits that elevated ICP leads to reduced perfusion of the optic nerve axons. The retrolaminar region of the optic nerve represents a vascular watershed zone and is therefore particularly susceptible to ischemic injury. Marked dilation of the distal optic SAS may compress both neural and vascular structures, compromising blood flow. Trobe proposed that papilledema arises from a competitive relationship between the posterior ciliary arterial circulation and the choroidal circulation. As subarachnoid pressure increases, the ciliary circulation becomes compressed, reducing arterial supply to the laminar optic nerve and interfering with the high-flow demands of the choroid (23). Chronic ischemia subsequently disrupts metabolic axoplasmic transport, resulting in axonal distension and accumulation of potentially toxic CSF metabolites (13).

Venous congestion constitutes an additional pathophysiological stressor: elevated retrolaminar pressure reduces venous outflow through the central retinal vein and peripapillary venous system, worsening tissue edema and disc swelling (22). The abrupt termination of the SAS at the lamina cribrosa magnifies these biomechanical effects, making even modest increases in ICP capable of producing substantial laminar deformation. In addition to translaminar pressure, the biomechanical properties of the lamina cribrosa and optic nerve head influence axonal damage induced by compression and/or ischemia. Without adequate control of ICP, prolonged compression ultimately results in irreversible axonal degeneration, thinning of the retinal nerve fiber layer (RNFL), and optic atrophy (21).

Given the continuity between cranial and ONSAS, the optic nerve sheath expands in response to rising ICP. Optic nerve sheath diameter (ONSD) measured 3–5 mm posterior to the globe—its most distensible region—has emerged as a useful non-invasive indicator of elevated ICP in adults and children (25). Sheath distension depends not only on instantaneous CSF pressure but also on tissue compliance and the mechanical properties of the trabecular and septal structures within the sheath. Chronic ICP elevation may alter these viscoelastic properties, potentially affecting serial measurements. In pediatric patients, developmental differences in tissue elasticity must be considered, although the coupling between ICP and ONSD appears preserved across age groups (26).

## 4 Key ophthalmologic findings in pediatric increased intracranial pressure

An increase in ICP can lead to significant ophthalmologic manifestations. The most prominent and clinically important ophthalmologic finding is papilledema, which involves swelling of the optic disc due to impaired venous drainage from the retina. Other common ophthalmologic signs include optic nerve sheath distension, which reflects the expansion of the SAS surrounding the optic nerve due to increased ICP, and vertical tortuosity of the optic nerve, which is thought to result from tension on the nerve caused by the increased pressure. Additionally, patients may show flattening of the posterior globe and choroidal folds, which are secondary to the alteration of ocular pressure dynamics (27).

Papilledema refers to optic disc swelling associated with ICP (13). Although its pathophysiology is not fully understood, several studies have established a clear relationship between elevated ICP and the development of papilledema. Its clinical importance as an indicator of raised ICP has long been recognized. Current evidence supports the concept that papilledema results from increased ICP transmitted to the SAS surrounding the optic nerve, disrupting normal axonal metabolism and leading to edema, ischemia, and potentially irreversible visual impairment or loss (11). Notably, not all individuals with elevated ICP develop papilledema, suggesting the presence of protective anatomical or physiological factors in the optic canal (28). Variability in the size and configuration of these spaces may modulate the degree to which ICP is transmitted to the optic nerve, thereby influencing axoplasmic flow stasis and the subsequent development of papilledema over a period of days (21, 29). In addition to its diagnostic value, papilledema may serve as a marker of disease severity and response to therapeutic interventions (30–34).

Papilledema is typically bilateral, although unilateral cases have been reported (35–37). The Frisén grading scale, a validated system, grades optic disc edema from 0, indicating no edema, to 5, indicating severe edema with loss of vessel architecture at the disc head (Table 1) (38). The degree of optic disc swelling correlates poorly with measured ICP and does not reliably predict the severity of clinical symptoms (11). Clinicians should also be aware that papilledema may be asymmetric (39). The Idiopathic Intracranial Hypertension Treatment Trial (IIHTT) reported that 7% of patients exhibited an interocular difference of two or more Frisén grades (38, 40). However, papilledema severity was identical between eyes in 58.2% of patients and within one grade in 92.8%, supporting the concept that papilledema is generally, though not invariably, symmetric (15).

**Table 1:** Frisén grading scale for optic disc swelling.

Stage	Nasal blur	Temporal blur	TOSV	Filled cup	Dome shape
0	-	-	-	-	-
1	+	-	-	-	-
2	++	+	-	-	-
3	+++	++	+	-	-
4	+++	+++	±	+	-
5	+++	+++	±	+	+

TOSV, total obscuration of segments of one or more major vessels outside the pole areas.

A subset of patients with symptoms suggestive of idiopathic intracranial hypertension (IIH) may have elevated opening pressure in the absence of papilledema. Up to 18% of such patients have been reported to lack papilledema despite elevated ICP (41, 42). In a large retrospective study of 353 patients with IIH, only 5.7% presented without papilledema (33). Similarly, Aylward et al. (41) reported that 17.7% of patients with elevated ICP did not exhibit papilledema. Glatstein et al. (43) identified elevated ICP without papilledema in 19.0% of patients aged between 2 and 16.5 years presenting to the emergency department (44).

The presence of spontaneous venous pulsations (SVP) has traditionally been considered evidence against elevated ICP. SVP are rhythmic variations in the caliber of the retinal veins, typically observed at or near the optic disc during fundoscopic examination. They result from the normal pressure gradient between IOP and ICP. SVP are present in approximately 80%–90% of healthy individuals, thus being a strong indicator of normal or non-elevated ICP. However, the absence of SVP is not, by itself, pathological and does not automatically imply raised ICP, since 20% of healthy individuals never display SVP.

Retinal manifestations associated with adult increased ICP are well described, but data in pediatric populations remain limited. Gospe et al. (45) evaluated 31 pediatric patients with IIH and found evidence of photoreceptor damage in 19% of eyes using optical coherence tomography (OCT), which was significantly associated with permanent vision loss. Curley et al. (46) conducted a retrospective analysis of 253 pediatric patients with increased ICP using fundus photography and optical coherence tomography. Retinal changes were identified in 9.5% of patients at presentation and included retinal folds (83%), macular exudates (63%), subretinal fluid (46%), and choroidal neovascular membrane (4%). The mean Frisén grade at presentation was 3.58 ± 0.96. Patients with retinal pathology were significantly older at diagnosis and demonstrated worse visual outcomes. After resolution of papilledema, optic atrophy occurred in 58% of patients with retinal findings compared with 5.2% of those without. Persistent structural retinal changes and outer retinal damage following papilledema resolution have been demonstrated in pediatric patients, similar to adult populations (45, 47). Retinal pathology at any point during the disease course was associated with worse visual function and a higher likelihood of optic atrophy. Subretinal fluid has been specifically associated with severe papilledema, possibly related to contiguous spread of optic disc edema (22).

Recent studies have clarified the pattern of retinal hemorrhages associated with isolated elevated ICP. Binenbaum et al. (48) identified retinal hemorrhages in 16% of children with nontraumatic elevated ICP, exclusively in the presence of optic disc swelling. These hemorrhages were superficial, intraretinal, and confined to the peripapillary region. Shi et al. (49) similarly concluded that retinal hemorrhages rarely occur in the absence of optic disc swelling and do not extend beyond the peripapillary area. When retinal hemorrhages are extensive, multilayered, or located away from the swollen optic disc, alternative etiologies such as trauma should be considered (48).

In the IIHTT, nerve fiber layer hemorrhages were observed in 27.2% of participants and correlated with papilledema severity. Optic disc hemorrhages were more frequent in patients who experienced treatment failure, suggesting a possible association with worse visual outcomes, although interpretation is limited by small sample size and restrictive inclusion criteria (50). Optic disc hemorrhages and cotton wool spots were associated with higher Frisén grades and worse visual function at presentation but did not independently predict final visual outcome (51, 52).

Diplopia is a recognized symptom in patients with increased ICP, with reported frequencies ranging from 12% to 60% in pediatric populations (35, 53, 54). The IIHTT reported diplopia in 18% of patients (40), while Guiseffi et al. (55) reported a higher incidence of 38%. Beyond papilledema, sixth cranial nerve (CN VI) palsy is the most common neurological finding in pediatric increased ICP (56, 57) and is considered the most frequent cause of diplopia, with one study reporting 14 cases in a cohort of 101 patients (58). Affected patients typically experience binocular horizontal diplopia, esotropia greater at distance than near, and abduction deficit (29).

The vulnerability of CN VI is attributed to its long intracranial course, including its relationship to the petrous temporal bone and its ascent between the pons and clivus before entering Dorello's canal. This anatomy renders the nerve susceptible to compression and stretching in the setting of raised ICP. Other cranial nerve palsies (III, IV and VII) have been reported but are rare (59). Reid et al. (60) suggest an incidence of CN VI palsy in pediatric patients with increased ICP of approximately 12%. CN VI palsy is often associated with papilledema and typically resolves completely with treatment, although recovery depends on the etiology and severity. Patients with CN VI palsy tend to have higher body mass index and opening pressure, although the role of these factors requires further investigation.

Optic disc edema without increased ICP may result from optic neuritis, neuroretinitis, anterior ischemic optic neuropathy, or infiltration of the optic nerve by tumor cells. In addition, pseudopapilledema may be caused by anomalous optic nerves, myelinated nerve fiber layers, or optic nerve drusen. These entities should be carefully considered, particularly in cases of unilateral or markedly asymmetric optic disc elevation (29).

## 5 Ophthalmologic diagnostic techniques and findings in increased intracranial pressure

### 5.1 Ophthalmic exam and fundoscopy findings

The ophthalmic examination is a critical component in the diagnosis and subsequent management of increased ICP. Key elements include assessment of visual acuity, pupillary responses, ocular motility, color vision, and a detailed funduscopic evaluation with particular attention to the optic nerve. A dilated fundus examination allows characterization of the optic nerve head appearance, and the presence and degree of optic nerve edema are essential for guiding diagnosis, follow-up, and therapeutic monitoring (42). Humphrey or Goldmann visual field testing, OCT of the RNFL, and fundus autofluorescence are helpful modalities for both initial evaluation and longitudinal monitoring (61). These techniques permit objective assessment of functional and structural changes associated with increased ICP (38, 62).

In cases of low-grade or early increased ICP, visual function often remains preserved for a prolonged period. This contrasts with other optic neuropathies, in which visual acuity, color vision, and visual fields are typically affected early in the disease course. As a result, normal findings on visual function testing do not exclude the presence of increased ICP, particularly in the early stages. In addition, it may appear later on, even after resolution of papilledema. This underscores the importance of careful funduscopic evaluation and adjunctive imaging techniques, as reliance on visual function alone may lead to under diagnosis or delayed recognition of papilledema in pediatric patients (63, 64).

In infants and young children, additional neuro-ophthalmologic signs may provide important diagnostic clues in the absence of overt papilledema. One such manifestation is tonic downgaze, characterized by a sustained downward deviation of the eyes, sometimes accompanied by eyelid retraction. This finding has been associated with increased ICP and may reflect dysfunction of supranuclear gaze pathways. Importantly, tonic downgaze can be present even in the absence of funduscopic abnormalities, particularly in younger patients, and should therefore raise clinical suspicion for elevated ICP in the appropriate context (65).

Funduscopic examination offers a noninvasive method for identifying papilledema and should be performed to assess for the presence of edema and graded using the Frisén scale. Papilledema is a highly specific indicator (98%) of elevated ICP, although its sensitivity varies with age. Papilledema is 100% sensitive in children older than eight years but indicates elevated ICP in only 22% of younger patients. These findings suggest that invasive ICP monitoring may be unnecessary in older children with detailed ophthalmologic examinations, whereas in younger children, absence of papilledema does not exclude elevated ICP (66). Sensitivity and specificity for detecting increased ICP are also limited by factors such as the timing of pressure development, chronicity of disease, and the anatomical location of underlying pathology (67).

Furthermore, in pediatric patients aged three to five years, papilledema is a strong predictor of permanent visual deficits, with approximately 50% of affected eyes experiencing some degree of permanent vision loss (45). The absence of hyperemia, hemorrhages, and cotton wool spots from the Frisén grading scale is intentional, reflecting variability across disease processes. Nevertheless, documenting these features in individual cases improves recognition of underlying etiology and temporal changes (38). Papilledema typically resolves within 4.2–5 months following normalization of ICP (68).

Papilledema presents with mechanical and vascular signs including optic nerve head elevation, blurring of disc margins and retinal vessels, venous engorgement, and capillary leakage (69). Carta et al. (70) identified four fundoscopic features with the greatest diagnostic accuracy: thickening of the peripapillary RNFL, peripapillary hemorrhages, anterior elevation of the optic nerve head, and congestion of arcuate and peripapillary venous vessels. The combination of these findings achieved an accuracy of 93%, sensitivity of 95%, and specificity of 89%.

The presence of SVP has traditionally been regarded as indicative of normal ICP, as SVP at the optic nerve may cease with increased ICP. However, these pulsations are present in only 87%–90% of normal individuals and may also occur in patients with documented ICP elevation. Therefore, diagnostic and therapeutic decisions should not rely on the presence or absence of SVP alone (29). Wong et al. (71) demonstrated visible SVP in 12 of 20 patients with increased ICP and mild papilledema, including five individuals with opening pressures exceeding 30 cmH₂O. Although limited by small sample size, this finding underscores that SVP does not reliably exclude raised ICP (39).

Pseudopapilledema caused by hyperopia or optic disc drusen can closely resemble papilledema due to elevated ICP, making differentiation a frequent source of neuro-ophthalmology referral in pediatric and adult populations (Table 2) (72). Diagnostic challenges arise because many patients with increased ICP may be asymptomatic, and headache features may be similar regardless of pressure status. In particular, unrecognized or undercorrected hyperopia represents a common and often underestimated cause of pseudopapilledema in children. A small, crowded optic disc associated with short axial length can mimic true optic disc edema, especially in the pediatric population, in whom physiologic hyperopia is frequent. Careful assessment of refractive error is therefore essential, and a detailed ophthalmologic examination under cycloplegia is critical to accurately identify hyperopia and avoid misdiagnosis. Cycloplegic refraction allows for precise evaluation of the true refractive status, prevents overestimation of optic disc swelling, and plays a pivotal role in distinguishing pseudopapilledema from papilledema secondary to elevated ICP.

**Table 2:** Differences between papilledema and pseudopapilledema.

Feature	Papilledema	Pseudopapilledema
Symptoms	Often headache, nausea, vomiting, transient visual obscurations, diplopia; may be subtle in young children	Often asymptomatic or non-specific headache
Laterality	Typically bilateral, may be asymmetric)	Often bilateral, can appear asymmetric
Disc appearance	Hyperemic swollen disc, blurred margins with vessel obscuration, possible hemorrhages and exudates	Elevated “lumpy-bumpy” disc, irregular margins, less true vessel obscuration, anomalous branching
Spontaneous venous pulsation	Often absent (not fully reliable)	May be present or absent (not decisive)
OCT	Marked RNFL thickening, optic nerve head elevation, subretinal/peripapillary hyporeflective space	RNFL may be mildly thick or with preserved architecture
FAF	Usually negative for drusen	Often positive in superficial drusen, may be negative if buried
B-scan ultrasound	ONSD may be enlarged; no calcified drusen	Calcified drusen may be visible, ONSD usually normal
Fluorescein angiography	Disc leakage	Late staining or minimal leakage
Visual fields	Enlarged blind spot, progressive defects if severe or chronic	Variable, arcuate or altitudinal defects are possible over time

OCT, optical coherence tomography; RNFL, retinal nerve fiber layer; FAF, fundus autofluoresence, ONSD, optic nerve sheath diameter.

Optic nerve head drusen may coexist with papilledema, necessitating careful evaluation. True optic nerve swelling is typically associated with blurred disc margins, vessel obscuration at the disc edge, and peripapillary hemorrhages or exudates. In contrast, optic disc drusen may present with irregular disc margins in asymptomatic individuals (73). Drusen are usually deeper during childhood and become more superficial with age.

Retinal and choroidal folds were considered pathognomonic for papilledema because they were not observed in pseudopapilledema, although sensitivity was low (23%). These findings are less reliable in pediatric patients, as buried optic disc drusen may also be associated with optic nerve head elevation, hemorrhages, and vascular abnormalities. Fundoscopic signs of optic disc drusen include excessive branching and coiling of retinal vessels and an irregular disc surface (74).

### 5.2 Optical coherence tomography and optical coherence tomography angiography

OCT is a non-invasive, high-resolution imaging technique that employs low-coherence interferometry to obtain detailed cross-sectional views of the retina, optic nerve head, and peripapillary structures. Conceptually, OCT functions similarly to an “optical ultrasound”; instead of sound waves, it uses near-infrared light to measure the time delay and intensity of reflected signals from retinal microstructures. These signals are then reconstructed to generate tomographic images with micrometer-level axial resolution, providing precise visualization of retinal and optic nerve architecture, among others. OCT has transformed the structural evaluation of both the anterior and posterior segments of the eye and is now regarded as an indispensable diagnostic tool in ophthalmology. Its capacity to produce real-time, fast (seconds) and *in situ* images without contact or tissue manipulation is particularly advantageous in clinical contexts where invasive assessment or direct histological sampling is neither practical nor safe (75).

**Figure 1:**
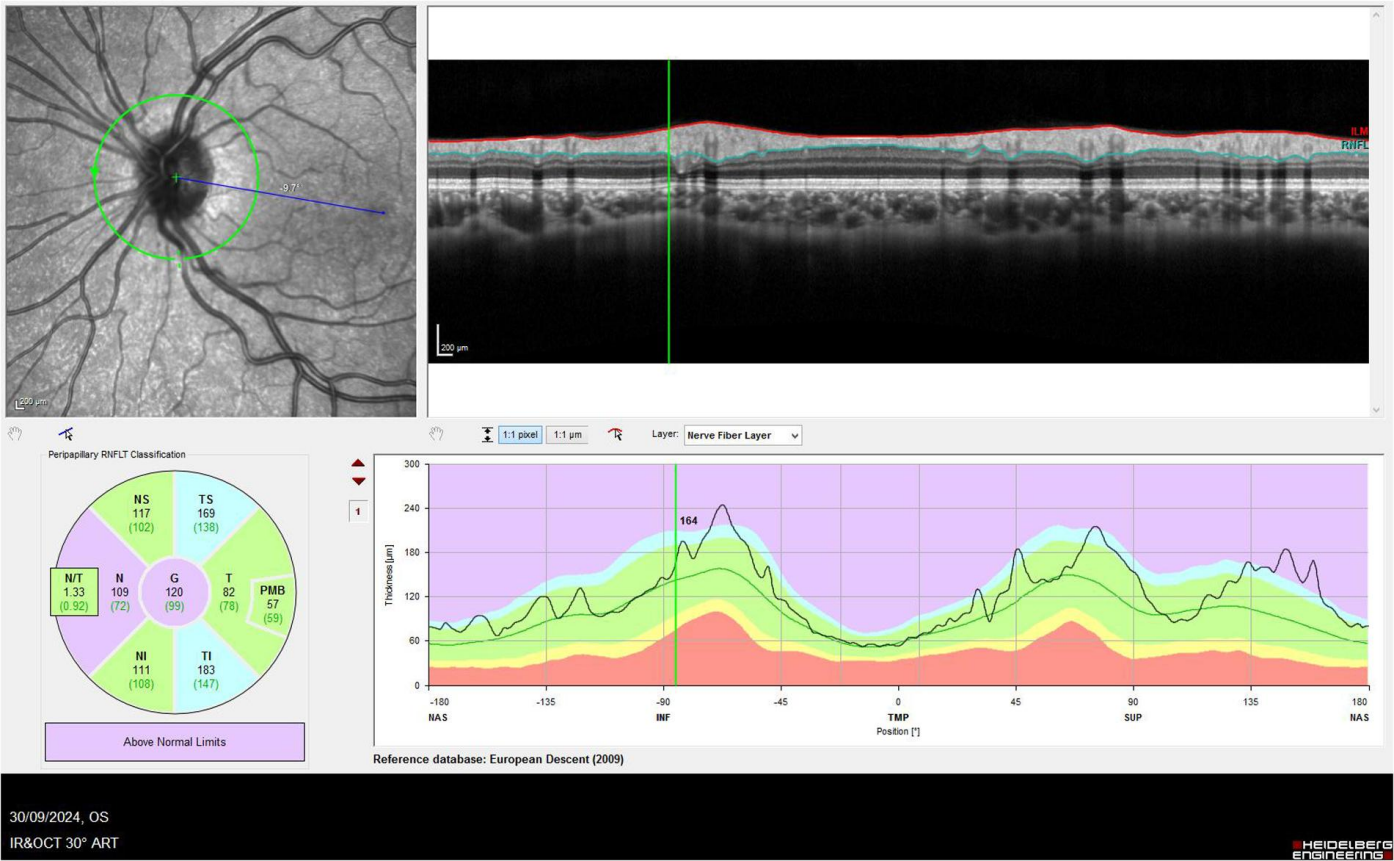
Optical coherence tomography of the optic nerve of a patient with mild papilledema. Analysis of the retinal nerve fibre layer shows an increase above normal values.

OCT provides quantitative information on optic nerve head morphology, including peripapillary RNFL thickening, deformation of Bruch's membrane (inward angulation), optic nerve head elevation, and the presence of subretinal or peripapillary hyporeflective spaces (Figure 1) (75, 76). OCT can also detect secondary features such as macular edema or peripapillary wrinkles, which may support a diagnosis of raised ICP (77, 78). Moreover, these changes have shown to correlate with both the severity and temporal course of raised ICP (79, 80). Clinically, OCT is used for both diagnosis and follow-up. RNFL measurements may detect incipient swelling even before it becomes ophthalmoscopically evident, while serial imaging allows clinicians to track changes after therapeutic interventions aimed at reducing ICP (76). Therefore, OCT findings complement the clinical examination and are especially valuable when optic disc swelling is subtle or in its early stages. Despite these advantages, several limitations affect its use in pediatric neuro-ophthalmology. Significant disc edema can distort retinal architecture, leading to segmentation errors that compromise RNFL and ganglion cell layer measurements; manual correction is often required and may not fully resolve inaccuracies. Interpretation is further complicated by physiological variability: RNFL thickness depends on age, axial length, refractive error, and ethnicity, and robust pediatric normative datasets remain limited (81). In chronic or mild ICP elevation, OCT findings may normalize despite ongoing functional compromise (82). Moreover, obtaining reliable scans in young children can be challenging due to reduced cooperation, motion artifacts, and the occasional need for sedation. Finally, OCT has also become a helpful tool for differentiating true papilledema from conditions that mimic optic disc swelling in children. Pseudopapilledema caused by optic disc drusen or congenitally crowded discs generally shows preserved retinal architecture, stable RNFL measurements, lack of intraretinal or subretinal fluid and Bruch's membrane maintains a normal contour (73, 83). Enhanced-depth imaging (EDI) OCT can further reveal buried optic disc drusen as hyperreflective ovoid structures beneath the surface of the optic nerve head, confirming the diagnosis when fundus examination is ambiguous (84).

Over the past decade, OCT angiography (OCT-A) has become an important complement to structural OCT, providing additional insight into microvascular alterations that cannot be appreciated on conventional imaging. OCT-A enables non-invasive visualization of the retinal and choroidal microvasculature by detecting motion contrast produced by erythrocytes as they flow through vessels. In contrast to traditional dye-based angiographic techniques such as fluorescein angiography, OCT-A does not require intravenous contrast administration. Instead, it relies on the acquisition of multiple sequential OCT B-scans at the same retinal location and identifies variations in signal amplitude or phase between scans, thereby differentiating perfused vasculature from static surrounding tissue (85). The method generates both en-face and cross-sectional representations of the retinal and choroidal circulation and allows segmentation of distinct vascular layers. This layered visualization facilitates precise quantification of vascular density, detection of flow deficits, and assessment of structural–vascular relationships that are not discernible with structural OCT alone (85).

OCT-A has emerging utility in the evaluation of optic nerve head perfusion and microvascular changes associated with elevated ICP (85). Studies have shown that alterations in peripapillary vessel density and flow indices may correlate with the degree of papilledema or optic nerve dysfunction, suggesting a potential role for OCT-A as an adjunctive biomarker of ICP-related pathology (86). Its rapid acquisition time, absence of contrast media, and high spatial resolution make it particularly appealing for use in children, who often have limited tolerance for invasive procedures. Several studies have demonstrated a correlation between peripapillary capillary density, particularly skeletonized vessel density and the severity of papilledema, RNFL thickness, and ganglion cell loss (86, 87). Reduced peripapillary capillary density may reflect microvascular compromise related to elevated ICP and can provide additional insights when structural findings are equivocal.

In contrast, fluorescein angiography remains the most reliable technique for differentiating these entities, as true papilledema characteristically demonstrates optic disc leakage, whereas pseudopapilledema does not. Importantly, in children, fluorescein can be administered orally, allowing delayed-phase imaging that still reveals disc leakage while avoiding intravenous access. Therefore, although OCT-A can add supportive microvascular information when used alongside structural OCT, it should be interpreted with caution and not considered a substitute for fluorescein angiography. Furthermore, its application in children is limited by motion artifacts and the need for patient cooperation during image acquisition, which may compromise image quality and reproducibility.

In the differential diagnosis between papilledema and pseudopapilledema (e.g., optic disc drusen or congenitally crowded discs), OCT-A may offer complementary diagnostic value, although its clinical utility remains a matter of debate. While true papilledema has been associated with microvascular dilation, increased flow signal in the peripapillary plexus, or capillary dropout in advanced cases, these findings are not sufficiently specific or consistent to reliably distinguish it from pseudopapiledema, particularly in pediatric populations (88). In contrast, fluorescein angiography remains the most reliable technique for differentiating these entities, as true papilledema characteristically demonstrates optic disc leakage, whereas pseudopapilledema does not. Importantly, in children, fluorescein can be administered orally, allowing delayed-phase imaging that still reveals disc leakage while avoiding intravenous access. Therefore, although OCT-A can add supportive microvascular information when used alongside structural OCT, it should be interpreted with caution and not considered a substitute for fluorescein angiography. Furthermore, its application in children is limited by motion artifacts and the need for patient cooperation during image acquisition, which may compromise image quality and reproducibility (89).

### 5.3 Intraocular pressure

IOP is a fundamental physiological parameter that reflects the balance between aqueous humor production and outflow within the eye. It plays a critical role in maintaining the structural integrity and optical properties of the globe, while deviations from normal levels are strongly associated with the development and progression of optic neuropathies, particularly glaucoma. IOP is primarily regulated by aqueous humor dynamics involving the ciliary body, trabecular meshwork, and uveoscleral pathways, and is influenced by both ocular and systemic factors. Beyond its local effects, IOP exists within a broader pressure environment and interacts with ICP at the level of the optic nerve head, where the TLPD has emerged as a key determinant of optic nerve structure and function.

Accurate measurement of IOP remains a cornerstone of ophthalmic evaluation. Normal IOP values at birth are lower than those observed in healthy adults. The mean IOP at birth is 8.4 ± 0.6 mmHg, with minimal variation during the first year of life (90). However, a physiological increase in IOP occurs with age (see Table 3). According to Pensiero et al., (90) IOP rises to 9.8 ± 0.4 mmHg after the first year of life and reaches 11.75 ± 0.6 mmHg from the age of five years onwards.

**Table 3:** Normal intraocular pressure (IOP) values from birth throughout childhood are summarized.

Age (years)	Pensiero et al. (90)	Goethals et al. (163)	Muir et al. (164)
	Mean ± SD (mmHg)	Mean ± SD (mmHg)	Mean ± SD (mmHg)
	*Non contact tonometer*	*Goldmann/Tonopen*	*Goldmann/Tonopen*
Birth	9.59 ± 2.3	–	10.0 ± 2.5
0–1	10.61 ± 3.1	7.8 ± 0.4	11.0 ± 2.2
1–2	12.03 ± 3.19	8.7 ± 0.6	11.8 ± 2.3
2–3	12.58 ± 1.6	9.5 ± 0.5	12.3 ± 2.0
3–4	13.37 ± 2.05	10.4 ± 0.3	12.9 ± 2.1
4–5	13.6 ± 2	11.7 ± 0.6	13.1 ± 2.0
5–6	14.1 ± 1.99	–	13.5 ± 2.1
6–7	14.51 ± 2.32	–	13.9 ± 2.2
7–8	14.95 ± 2.29	–	14.4 ± 2.2
8–9	14.75 ± 2.92	–	14.7 ± 2.1
9–10	14.97 ± 2.25	–	15.0 ± 2.2
10–11	13.72 ± 2.10	–	15.1 ± 2.1
11–12	13.97 ± 2.42	–	15.3 ± 2.0
12–13	14.89 ± 1.98	–	15.5 ± 2.1
13–14	13.94 ± 1.78	–	15.7 ± 2.1
14–15	14.09 ± 2.47	–	15.8 ± 2.0

SD, standard deviation; mmHg, millimeters of mercury.

Note that the IOP values increase with age, mainly from the fifth year of life onwards.

In non-cooperative patients, examination is performed under anesthesia, and in this context IOP measurements may be influenced by the effects of anesthetic agents. Ketamine, used historically, produces an increase in IOP, whereas halothane, sevoflurane, and desflurane lead to a reduction in IOP (90, 91). Currently, sevoflurane is the most commonly used agent, providing a more stable and predictable decrease in IOP.

Goldmann applanation tonometry (GAT) remains the gold standard for IOP measurement, and its portable version—the Perkins tonometer—is the device most commonly used in pediatric patients, either during examination under anesthesia or in the outpatient setting in cooperative children. However, it presents several limitations that can affect its accuracy and applicability. GAT requires direct corneal contact, precise alignment, and the use of topical anesthesia, which can be challenging in uncooperative patients, including young children. Its accuracy is strongly influenced by corneal properties such as central corneal thickness, curvature, rigidity, and the presence of edema, scars, or irregular astigmatism. These factors limit its utility in pediatric populations and in patients with significant corneal abnormalities, making alternative methods necessary in these contexts.

In recent years, new tonometric approaches have become available that may prove useful in this patient population, including rebound tonometry (RT), air-pulse tonometry, and non-contact tonometry (NCT).

RT has emerged as an effective and widely accepted substitute for GAT. Its design, based on a small magnetized probe that briefly contacts and rebounds from the corneal surface, allows estimation of IOP by analyzing the deceleration profile of the probe. Owing to several practical advantages over GAT—such as rapid acquisition, ease of use, minimal contact area, and the absence of a requirement for topical anesthesia—its use has expanded considerably in clinical practice, particularly in pediatric populations, where it is now considered the first-line option for screening in many settings. Although RT instruments tend to overestimate IOP relative to GAT, measurements remain largely interchangeable when IOP is lower than 19 mmHg (92–95).

NCT, which uses an air pulse to applanate the cornea without direct contact, represents a useful alternative for measuring IOP in school-aged children. Its main advantages are the absence of topical anesthesia and its completely non-invasive nature, which improves tolerance in cooperative patients. Although accuracy has improved with newer devices, measurements still tend to show greater variability and a slight overestimation of IOP compared with GAT, with a mean overestimation of +1 to +3 mmHg and larger discrepancies in eyes with high IOP or increased corneal thickness.

More recent devices, such as the Ocular Response Analyzer (ORA) and Corvis ST—also referred to as advanced air-puff tonometers—allow not only the measurement of IOP but also the assessment of corneal biomechanics (96, 97). The Corvis ST combines an air pulse with a high-speed Scheimpflug camera to capture the full dynamic corneal deformation response. It records the entire deformation process, generating several advanced biomechanical indices, including the biomechanically corrected IOP. It has demonstrated greater accuracy than conventional NCT when compared with GAT.

Recently, the experimental study by Zhu et al. (98) investigated in an *in vivo* model how IOP and ICP interact significantly in the biomechanics of the optic nerve. The authors demonstrated that both variables generate significant and distinct effects on the structural deformation of optic nerve tissues, including displacement, curvature, and laminar strain. Increased ICP was associated with posterior–anterior displacement of the lamina cribrosa and changes in its configuration, supporting a functional biomechanical relationship between IOP and ICP. Although this physiological interdependence is clear, the degree to which IOP reflects changes in ICP varies between individuals, limiting its clinical usefulness as a direct surrogate for intracranial measurement.

A moderate to high positive correlation between IOP and ICP has been described in adult patients presenting with acute increases in ICP. Lashutka et al. (99) reported high sensitivity and specificity of elevated IOP for detecting pathological ICP; in a pioneering study of 27 patients with invasive ICP monitoring and IOP measured using a handheld tonometer, elevated ICP was correctly identified with 100% sensitivity and specificity. This author supports the potential utility of IOP as a non-invasive indicator of altered ICP in emergency and neuro-trauma settings.

However, most authors agree that although a statistically significant correlation exists between IOP and ICP, the discriminatory capacity of IOP as a diagnostic method for increased ICP is low. Li et al. (100), analyzing a cohort of 130 patients in whom ICP was measured by lumbar puncture and IOP by Goldmann tonometry, found a significant correlation between both pressures. However, the accuracy for predicting ICP value was only 65.4%. Conversely, Golan et al. (101) concluded that handheld tonometry is a poor screening tool for elevated ICP in neurological patients, with low ability to discriminate between elevated and normal ICP. Similarly, the meta-analysis by Yavin et al. (102) found a moderate overall correlation (r ≈ 0.5–0.6) between IOP and ICP, but insufficient sensitivity and specificity to use IOP as a standalone diagnostic tool for elevated ICP.

In pediatrics, findings are somewhat more favorable but still limited. Spentzas et al. (103) studied 36 children with severe traumatic brain injury and invasive ICP monitoring, performing 274 simultaneous IOP measurements. They found good statistical agreement between IOP and ICP and, using a cutoff of 20 cm H₂O for normal ICP, IOP demonstrated a sensitivity of 0.96–0.97 and specificity of 0.70–0.78 for detecting increased ICP. Other studies in children with hydrocephalus or various causes of increased ICP have reported marked variability in this association, and collectively agree that IOP cannot serve as a substitute for invasive monitoring (101, 104).

### 5.4 Visual evoked potentials

Visual evoked potentials (VEP) are non-invasive electrophysiological recordings that capture the brain's cortical response to visual stimulation. By measuring the electrical activity generated along the visual pathway from the retina and optic nerve through the chiasm and optic radiations to the occipital cortex. VEP provide an objective assessment of the functional integrity of these structures (105).

In clinical practice, surface electrodes are placed on the scalp over the occipital region while the patient views a series of patterned or flashing stimuli displayed on a monitor. The resulting signals are averaged and analyzed to identify characteristic waveforms that reflect the timing and amplitude of neuronal conduction within the pathway. VEP are particularly valuable in situations where conventional visual acuity assessment is unreliable or not feasible, including preverbal children, those with developmental delay, or patients unable to cooperate with subjective tests. Flash VEP, although less specific than pattern-reversal stimuli, are useful when fixation is unstable or absent and therefore have a practical role in infants and young children (105, 106).

In cases of raised ICP, VEPs have been investigated as a functional test to detect optic nerve dysfunction, such as slowed conduction velocity, increased latency, reduced amplitude, or abnormal wave morphology, which may reflect compression or ischemia of the optic nerve head or retrobulbar fibers secondary to papilledema, optic nerve swelling, or compromised axoplasmic flow (107). In pediatric populations, VEPs offer specific advantages: they are non-invasive, technically straightforward, and feasible in non-verbal, developmentally delayed, or uncooperative children, particularly with flash VEPs. VEP abnormalities may appear before structural damage becomes evident on funduscopy or OCT, making them a potentially sensitive tool for detecting early functional compromise in chronic or fluctuating increased ICP (108).

However, the limitations of VEP are substantial. Their results are influenced by refractive errors, media opacities, poor attention, variable alertness, and electrode placement, all of which reduce specificity, especially in younger children. Abnormal VEP parameters are not specific to elevated ICP and can occur in demyelinating disease, optic neuritis, hereditary optic neuropathies, or toxic-nutritional optic neuropathies (109). Moreover, VEPs lack anatomical specificity and cannot localize the site of dysfunction along the visual pathway. They provide no information regarding RNFL thickness, optic disc elevation, or other structural changes relevant to papilledema. As such, VEPs cannot replace neuroimaging or lumbar puncture, and neither a normal nor an abnormal VEP result allows confirmation or exclusion of raised ICP.

When interpreted alongside OCT, fundus examination, neuroimaging, and clinical findings, VEPs may provide useful supplementary data, particularly in children who cannot reliably perform subjective visual testing. Their role is supportive rather than diagnostic, and they should be used within a multimodal assessment framework.

### 5.5 Optic nerve sheath diameter ultrasound

The most extensively studied noninvasive technique for detecting elevated ICP is ocular ultrasound measurement of the ONSD. The optic nerve sheath is an extension of the dura mater, and the SAS extends from the intracranial compartment to surround the optic nerve beneath the sheath. Increases in ICP lead to expansion of the optic nerve sheath, which can be measured ultrasonographically. ONSD is typically measured 3 mm behind the papilla when ultrasound demonstrates a clear and uniform low-reflection band extending posteriorly from the globe, with parallel thin echo lines representing the sheath. Each eye should be measured in multiple planes and angles, and the values averaged. This technique has demonstrated good reproducibility, observer agreement, and measurement accuracy (110).

Ocular ultrasound has several limitations, including inter-rater variability and lack of standardization, requiring appropriate training and experience. Although the learning curve for ONSD measurement is relatively low and staff can be trained efficiently, interobserver variability contributes to false positive and false negative results (111). Multiple studies have shown high inter- and intraobserver variation in ONSD measurements, although these variations tend to decrease with increased experience and over time (26, 112).

Differences in examination technique further affect repeatability. Measurements obtained in different planes may vary significantly, and image quality is critical. Artifacts occur more frequently closer to the globe and may result in inaccurate measurements. In some cases, the measured structure may represent optic nerve shadowing rather than the sheath itself. Poor insonation angle, limited horizontal resolution, and ultrasonic blooming effects may introduce bias. Gain settings also influence measurements, with lower gain leading to apparent overestimation and higher gain resulting in smaller measured diameters (113, 114).

Patients with enlarged ONSD have been shown to face a greater risk of mortality compared with those with normal measurements. Given that bedside ultrasonographic ONSD assessment demonstrates high sensitivity and specificity, it may be used as a surrogate marker to guide therapy in patients at risk for elevated ICP (26).

There is substantial variability in proposed ONSD cutoff values across studies. Amini et al. (115) reported a strong correlation between ONSD and ICP (*r* = 0.88) and found that a cutoff of 5.5 mm yielded perfect sensitivity and specificity for predicting ICP above 15 mm Hg. Although several other studies reported sensitivities above 90%, none fully replicated these findings (116–118).

Variability in cutoff values, ranging from 4 to 6 mm, may reflect ethnic differences and developmental factors, as ONSD increases with age (119). Most studies adopt cutoff values of 4.5 mm or greater in children older than one year and 4.0 mm in children younger than one year. Serial measurements may assist in predicting increased ICP and guiding therapy in neurocritical care (26). While Ballantyne et al. (120) have proposed cutoffs of 4 mm for infants and 4.5 mm for older children as the limits of normal ONSD, these cutoffs may have low sensitivity based on data from children with elevated ICP and a measured ONSD (121).

ONSD correlates more strongly with ICP above 15 mmHg, limiting sensitivity for detecting changes within the normal or low-pressure range (115, 122). Historically, B-scan ultrasonography has been used to identify calcified optic nerve drusen and differentiate true papilledema from pseudopapilledema. In younger patients whose drusen are not calcified, fundus autofluorescence may serve as a useful adjunct (123, 124).

B-scan ultrasound can also be used to assess optic nerve sheath diameter. Although ultrasound cannot replace lumbar puncture, pediatric studies have shown that optic nerve sheath diameters exceeding 4.5 mm correlate with elevated opening pressure (using a cutoff of 20 cm H_2_O) (29, 125). Sensitivity is lower in children than adults, likely due to less calcification of drusen (126). Fundus autofluorescence and formal perimetry may provide additional diagnostic support. Fluorescein angiography demonstrates leakage in true papilledema and late staining in optic disc drusen and has shown high diagnostic accuracy for differentiation (73, 127, 128).

### 5.6 Neuroimaging

Neuroimaging plays a pivotal role in both the assessment of ICP and the identification of its underlying causes. Several neuroimaging modalities are used to assess ICP, including computed tomography (CT) and magnetic resonance imaging (MRI), which is considered the most comprehensive tool, especially when it comes to detailed visualization of brain structures, soft tissues, and vascular changes. In recent years, advancements in neuroimaging techniques, such as orbital MRI sequences, and the increased use of MRI angiography (MRA) and MRI venography (MRV), have enhanced the ability to visualize intracranial structures and vascular changes associated with elevated ICP.

One of the emerging techniques for evaluating elevated ICP is the use of orbital MRI sequences. These sequences specifically focus on the optic nerves and the retrobulbar area, which are sensitive to pressure changes in the cranial cavity. The optic nerves, as they travel through the optic canal, can show signs of increased ICP. The application of specialized orbital sequences allows for high-resolution visualization of the optic disc and the surrounding structures, providing valuable insight into optic nerve head edema and other signs of increased ICP (129).

Although there is no single definitive protocol, axial and coronal T1-weighted images without fat suppression, axial and coronal short-T1 inversion recovery (STIR) images, and axial and coronal post-contrast fat-suppressed T1-weighted images are common clinical MRI protocols for the optic nerve. MRI magnetic field strength of 1.5 T or 3 T is recommended, and the slice thickness should be less than 3 mm. The orbit and cavernous sinus should be included in both axial and coronal images (130).

Moreover, these sequences are non-invasive and can be used repeatedly to monitor ICP changes over time, particularly in patients who require continuous monitoring but cannot undergo invasive procedures like ICP monitoring. While orbital MRI offers excellent sensitivity in detecting optic nerve abnormalities, it is often used in conjunction with other imaging modalities for a more comprehensive assessment.

In addition to orbital sequences, standard brain MRI techniques are instrumental in detecting indirect signs of elevated ICP. These sequences provide detailed views of brain parenchyma, highlighting areas of edema, herniation, and compression of critical brain structures. Additionally, diffusion-weighted imaging and perfusion-weighted imaging are advanced MRI techniques that help assess ischemia and regional cerebral blood flow alterations, which are often associated with increased ICP.

MRA and MRV offer non-invasive methods to assess the vascular structures of the brain and their involvement in conditions associated with elevated ICP. MRA is particularly useful for visualizing arterial structures in the brain, enabling the detection of conditions such as stenosis, aneurysms, arteriovenous malformations, and other vascular abnormalities. It provides detailed images of the blood vessels without the need for contrast agents containing iodine, which can be beneficial in patients with renal insufficiency. MRV, on the other hand, is specifically designed to assess venous structures and is commonly used to evaluate venous thrombosis, cerebral venous sinus thrombosis, and abnormalities in venous drainage. Both MRA and MRV offer high-resolution images, allowing for accurate diagnosis and treatment planning (129).

One of the primary goals of neuroimaging in the context of elevated ICP is to detect direct signs of pressure increases, identify underlying causes, and monitor the effects on brain structures and vascular systems. Common neuroimaging findings in cases of elevated ICP include cerebral edema, midline shift of brain structures, indicating mass effects due to tumors, hemorrhages, or diffuse brain swelling, which is a key indicator of significant ICP elevation. Brain herniation is one of the most severe consequences of elevated ICP, referring to the downward or lateral displacement of brain tissue through openings such as the tentorial hiatus or foramen magnum, which represents a potentially life-threatening condition requiring immediate intervention (131).

**Figure 2:**
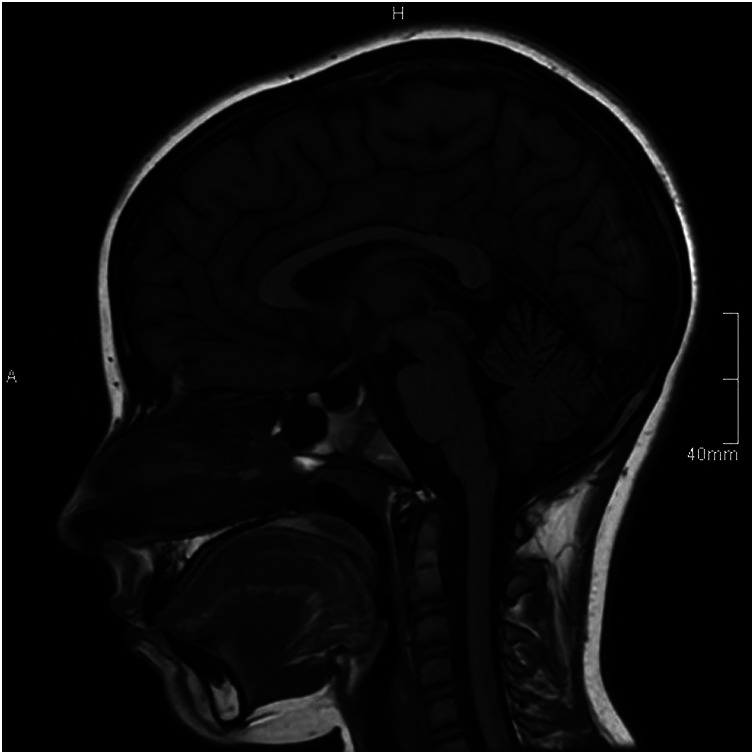
Sagittal T1 magnetic resonance imaging showing a partially empty sella filled with cerebrospinal fluid with a thin rim of pituitary tissue.

In the case of IIH, several neuroimaging findings are particularly relevant. One of the key findings in patients is a partially empty sella, seen in 85% of cases, although it is also quite common in the general population (Figure 2). This occurs when long-term increased ICP causes the arachnoid membrane to protrude through the diaphragmatic sella, leading to flattening of the pituitary gland. As a result, the top part of the pituitary gland becomes concave. This flattening happens because of a defect in the diaphragmatic sella, either congenital or acquired, which allows the arachnoid membrane and CSF to herniate through the opening due to the elevated ICP (129, 132).

Orbital findings reflect the mechanical changes in the optic nerve sheath, lamina cribrosa, and posterior sclera due to increased CSF pressure transmitted along the optic nerve. Posterior globe flattening and optic nerve head protrusion occur when the normal curvature of the posterior sclera is lost, visible on axial MRI where the optic nerve enters the eye. Interestingly, this finding can sometimes precede papilledema, highlighting its importance in early diagnosis. Tortuosity of the optic nerve can also be seen, which indicates that the nerve appears more twisted or curved due to pressure in its sheath. Vertical tortuosity is especially characteristic of idiopathic increased ICP (129).

MRI and MRV are essential tools for detecting narrowing of the venous sinuses, particularly the transverse sinuses, which is common in IIH. This stenosis can contribute to venous congestion and impaired absorption of CSF, exacerbating increased ICP. MRV can show focal stenosis of the transverse sinuses, while MRA is useful for visualizing abnormal arterial structures, stenosis, or vascular malformations that could contribute to elevated ICP. This technique allows for the evaluation of both the arterial and venous systems, providing crucial insights into conditions such as venous sinus thrombosis, which can worsen increased ICP due to altered venous drainage (129).

In pediatric patients, there are limited studies on the imaging findings and it is unclear whether the findings from adults can be applied to children. A study examining the optic nerve sheaths in pediatric patients with increased ICP found them to be larger compared to normal children. Another study found no significant differences between pediatric patients and controls except for vertical tortuosity of the optic nerves. However, a subsequent study reported that several neuroimaging signs, such as empty sella, flattening of the posterior globe, optic nerve head protrusion, optic nerve sheath distension, and optic nerve tortuosity, were more common in pediatric patients than in controls. Differences in study design, particularly the use of general anesthesia in some studies (which may have temporarily raised CSF pressure), could explain the disagreement. A comparison of two studies—one with 16 adults and one with 23 children—using the same imaging criteria showed no significant differences in neuroimaging findings between the two groups, except for the prevalence of empty sella, which was lower in children (26% vs. 69%, *P* = 0.01) (129). Hartman et al. (133) revealed that prepubescent children had significantly lower rates of scleral flattening, empty or partially empty sella, and transverse sinus stenosis compared to adolescents and adults. They also had lower, though not statistically significant, rates of perioptic increased CSF and optic nerve tortuosity. Adults had a significantly lower rate of optic nerve head enhancement compared to both prepubescent children and adolescents. There were no significant differences in optic nerve head protrusion, enlargement of Meckel's cave, or tonsillar herniation between groups.

Neuroimaging is a crucial tool in the diagnosis and management of elevated ICP. Although pediatric increased ICP presents some unique challenges, MRI remains the gold standard for diagnosing and monitoring the disease in children, as it does in adults. Pediatric neuroimaging typically includes orbital sequences to assess optic nerve findings and contrast-enhanced MRI to evaluate venous drainage.

### 5.7 Transcranial Doppler ultrasonography

Transcranial Doppler ultrasonography measures blood flow through major intracranial vessels and is traditionally used in the diagnosis of emboli, stenosis, and vasospasm. Several transcranial Doppler–based techniques have been proposed to estimate ICP. The most extensively studied approach involves the Gosling pulsatility index, calculated as the difference between peak systolic and end-diastolic velocity divided by mean flow velocity in the bilateral middle cerebral arteries (134).

Bellner et al. (135) reported a strong correlation between pulsatility index and ICP, with a correlation coefficient of 0.94. However, subsequent studies demonstrated weaker associations, often below 0.5, limiting the reliability of pulsatility index as an independent estimator of ICP (136). Other methods estimate ICP indirectly using cerebral perfusion pressure, mean arterial pressure, blood flow waveform characteristics, diastolic vs. mean flow, or critical closing pressure. Cardim et al. (137) prospectively compared four transcranial Doppler–based estimation methods and found that a combined estimator performed better than individual techniques, although overall correlation remained low (*r* = 0.47).

Two-depth transcranial Doppler uses applied extracranial pressure to estimate ICP. The principle is that blood flow waveforms in the extracranial and intracranial segments of the ophthalmic artery become equivalent when applied pressure equals ICP. This technique requires a headframe to stabilize the ultrasound probe and accurate application of orbital pressure (122).

Ragauskas et al. (138) used two-depth transcranial Doppler to estimate ICP in patients with predominantly normal pressures and reported a strong linear correlation (*r* = 0.8), with all measured values within ±4 mm Hg of invasive measurements. In contrast, Bershad et al. (139) reported weaker correlations (*r* = 0.51) and wider error ranges, with technical limitations preventing successful measurements in a substantial proportion of patients. Koskinen et al. (140) similarly reported wide confidence intervals and limited feasibility.

To date, MRI-ICP and two-depth transcranial Doppler are the only noninvasive techniques directly compared with invasive ICP measurements. Other noninvasive approaches rely primarily on correlations with secondary parameters, and it remains uncertain whether these correlations can be translated into accurate ICP estimation, underscoring the challenges of developing reliable noninvasive monitoring strategies (122, 141, 142).

### 5.8 Electroencephalogram

Electroencephalography (EEG) is a non-invasive diagnostic tool that records the electrical activity of the brain, offering valuable insights into a wide range of neurological disorders. The EEG can help identify patterns associated with cortical visual processing, as well as assist in the diagnosis of neuroophthalmological conditions.

EEG changes correlate with cerebral blood flow and levels of consciousness, which are related to ICP (143). Studies have examined relationships between EEG burst duration and ICP as well as temporal associations between EEG and pressure changes. Among these investigations, only Chen et al. (144) reported a correlation coefficient, demonstrating a negative correlation between an EEG-derived pressure index and ICP measured by lumbar puncture (*r* = −0.849).

### 5.9 Ophthalmodynamometry

In 1917, Bailliart described ophthalmodynamometry based on the premise that a retinal blood vessel collapses when ocular pressure equals or exceeds intravascular pressure (145). Since then, ophthalmodynamometry has been used in the evaluation of ocular vascular disorders, primarily arterial disease. A relationship between retinal venous pressure and ICP was later suggested by Baurmann (146).

In 1925, it was proposed that ICP was responsible for pulsation of the central retinal vein and that venous outflow pressure must be at least as high as ICP because the vein travels within the optic nerve sheath and is surrounded by cerebrospinal fluid (146). By increasing IOP until collapse of the central retinal vein is observed, venous outflow pressure can be estimated as a reflection of ICP.

Firsching et al. (147, 148) evaluated this technique by anesthetizing the eye, applying a suction cup to the lateral globe, and increasing ocular pressure stepwise until collapse of the central retinal vein was observed by funduscopy. A conversion chart was used to determine IOP, and venous outflow pressure was defined as the sum of ocular pressure and applied suction. In a cohort of 105 patients, this method demonstrated a sensitivity of 72.7% and a specificity of 96.2% for predicting ICP ≥15 mm Hg, with a correlation coefficient of *r* = 0.690.

Querfurth et al. (149) performed 22 measurements in six patients, evaluating venous outflow pressure in conjunction with central retinal and ophthalmic artery pulsatility indices. They reported a correlation of *r* = 0.87 between venous outflow pressure and ICP, which improved to *r* = 0.95 when ophthalmic artery pulsatility indices were included. The improved correlation may be partially explained by repeated measurements in the same patients rather than sampling a broader population.

Under normal physiological conditions, central retinal vein pressure is equal to or slightly higher than ICP, as cerebrospinal fluid surrounding the optic nerve sheath influences venous pressure before drainage into the cavernous sinus. Ophthalmodynamometry, traditionally used to assess arterial pressure, can also be applied to measure retinal venous pressure, providing a more objective assessment than indirect fundoscopic signs.

Two clinical studies recorded central retinal vein pressure in patients undergoing simultaneous invasive ICP monitoring, primarily for suspected hydrocephalus (147, 150). Normal venous outflow pressure values were below 20 mm Hg, whereas values above 30 mm Hg were associated with ICP greater than 15 mm Hg. Ophthalmodynamometry provides only momentary measurements and is unsuitable for continuous monitoring. However, it is noninvasive, repeatable, and may be helpful in the differential diagnosis of shunt malfunction, hydrocephalus, and brain atrophy (147). Both studies demonstrated strong linear correlations between retinal venous pressure and ICP (*r* ≈ 0.97), supporting ophthalmodynamometry as a practical adjunctive tool, although invasive techniques remain necessary for continuous ICP monitoring.

## 6 Clinical relevance and diagnostic workflow for intracranial pressure assessment in children

The diagnostic evaluation of suspected raised ICP in children requires a multimodal and context-dependent approach (Table 4), as no single ophthalmic or bedside modality provides sufficient standalone diagnostic certainty across all clinical scenarios. Fundoscopy remains an essential first-line examination, but the absence of papilledema does not reliably exclude elevated ICP, particularly in pediatric patients. Likewise, OCT and OCT-A offer valuable structural and microvascular information, especially when distinguishing papilledema from pseudopapilledema, yet they do not directly measure ICP and remain subject to age-related, technical, and interpretative limitations. Among non-invasive adjunctive tools, OSND ultrasound currently has the strongest pooled pediatric diagnostic performance, although its interpretation remains operator-dependent and threshold-sensitive. Neuroimaging and lumbar puncture therefore continue to occupy central roles in etiologic evaluation and diagnostic confirmation when clinically appropriate. Collectively, these considerations support a stepwise, integrated diagnostic strategy in which individual modalities are interpreted as complementary rather than interchangeable.

**Table 4:** Diagnostic tests for suspected raised intracranial pressure (ICP).

Modality	Reported diagnostic performance *	Main strengths	Key limitations	Clinical role in practice
**Fundoscopy/optic disc examination (**165, 166)	**Variable** ; absence of papilledema does **not** exclude raised ICP	First-line, universally relevant, non-invasive, essential component of bedside ophthalmic evaluation	Papilledema may be absent despite elevated ICP; subjective interpretation; interobserver variability; difficult in younger or uncooperative children; does not directly quantify ICP	**Initial screening/essential clinical examination** ; helps identify definite papilledema and guide urgency of work-up
**OCT (**167–169)	**Variable across studies** ; useful for structural characterization rather than direct ICP detection	Objective quantification of RNFL/optic nerve head changes; helpful in distinguishing papilledema from pseudopapilledema; useful for longitudinal follow-up	No universal pediatric cutoffs; device- and protocol-dependent; requires cooperation; not a direct ICP measure; may be limited by media opacity or motion artifact	**Adjunctive ophthalmic tool** for disc characterization and follow-up, especially when pseudopapilledema is a concern
**OCT-A (**88)	**Emerging/not yet standardized**	Non-invasive assessment of peripapillary microvasculature; may provide complementary information in equivocal disc swelling	Limited pediatric evidence; no validated thresholds; motion artifacts; requires cooperation; currently insufficient as standalone diagnostic modality	**Optional adjunct** in selected cases; primarily investigational or complementary
**OSND ultrasound (**170–173)	**Best-supported pooled pediatric performance among non-invasive adjuncts** ; pediatric meta-analytic estimates approximately **89%–92% sensitivity** and **72%–76% specificity**, depending on ICP threshold and methodology	Rapid, bedside, non-invasive, repeatable; useful in emergency, ICU, and resource-limited settings; may support suspicion when fundoscopy is delayed or equivocal	Operator-dependent; threshold variability by age and technique; indirect marker of ICP; lower specificity than sensitivity; should not replace etiologic imaging or lumbar puncture	**Bedside adjunctive screening/triage tool** to support suspicion of raised ICP
**Orbital ultrasound (B-scan/A-scan) (**174)	**Variable** ; more useful for **pseudopapilledema/optic disc drusen** than for direct ICP assessment	Can help identify optic disc drusen; relatively accessible; useful when disc elevation is equivocal	Limited value as a direct test for raised ICP; operator-dependent; performance varies by expertise and equipment	**Adjunctive differential tool** when distinguishing papilledema from pseudopapilledema
**Brain MRI and MRV (**166, 175)	**Not a direct ICP measurement** ; diagnostic yield depends on underlying etiology and presence of secondary signs	Identifies structural and vascular causes (mass lesion, hydrocephalus, CVST); central to etiologic evaluation; no ionizing radiation	May require sedation; not always rapidly available; indirect signs may be absent; cost and access limitations	**Core etiologic investigation** in suspected raised ICP, especially when red flags or papilledema are present
**Computed tomography (**166, 175)	**Useful for urgent exclusion of major structural causes** , but less sensitive than MRI for several etiologies	Rapid, widely available in emergencies; useful for acute hydrocephalus, hemorrhage, or mass effect	Radiation exposure; lower sensitivity than MRI for subtle or venous etiologies; not a direct ICP test	**Emergency alternative** when MRI is not immediately available
**Lumbar puncture (**166, 175)	**Reference standard for pressure confirmation when safely indicated**	Direct opening pressure assessment; provides CSF composition; central in diagnostic confirmation of intracranial hypertension in appropriate settings	Invasive; contraindicated in some settings; opening pressure influenced by positioning, sedation, agitation, obesity, and technical factors	**Confirmatory test** after neuroimaging excludes contraindications and when clinical suspicion persists
**Visual field testing (**166, 167)	**Supportive rather than diagnostic for ICP detection**	Functional assessment of visual involvement; useful for documenting disease burden and monitoring progression	Requires cooperation and reliability; limited in younger children; not diagnostic in isolation	**Monitoring/functional follow-up tool**
**Visual evoked potentials (**166)	**Adjunctive/non-specific**	May demonstrate optic pathway dysfunction in selected cases	Limited specificity; age-dependent variability; not useful as primary test for raised ICP	**Selective adjunct** in specific neuro-ophthalmic scenarios

OCT, optical coherence tomography; RNFL, retinal nerve fiber layer; OCT-A, OCT angiography; ONSD, optic nerve sheath diameter; MRI, magnetic resonance imaging; MRV, magnetic resonance venography; CSF, cerebrospinal fluid; CVST, cerebral venous sinus thrombosis.

* Reported diagnostic performance should be interpreted cautiously, as available pediatric studies are heterogeneous with respect to study design, reference standards, age distribution, disease spectrum, and diagnostic thresholds. Accordingly, values are presented as representative rather than directly comparable across modalities. Among non-invasive adjunctive tools, OSND ultrasound currently has the most reproducible pooled pediatric diagnostic accuracy estimates, whereas fundoscopy, OCT, and OCT-A remain highly clinically relevant but are less amenable to a single pooled performance metric.

**Figure 3:**
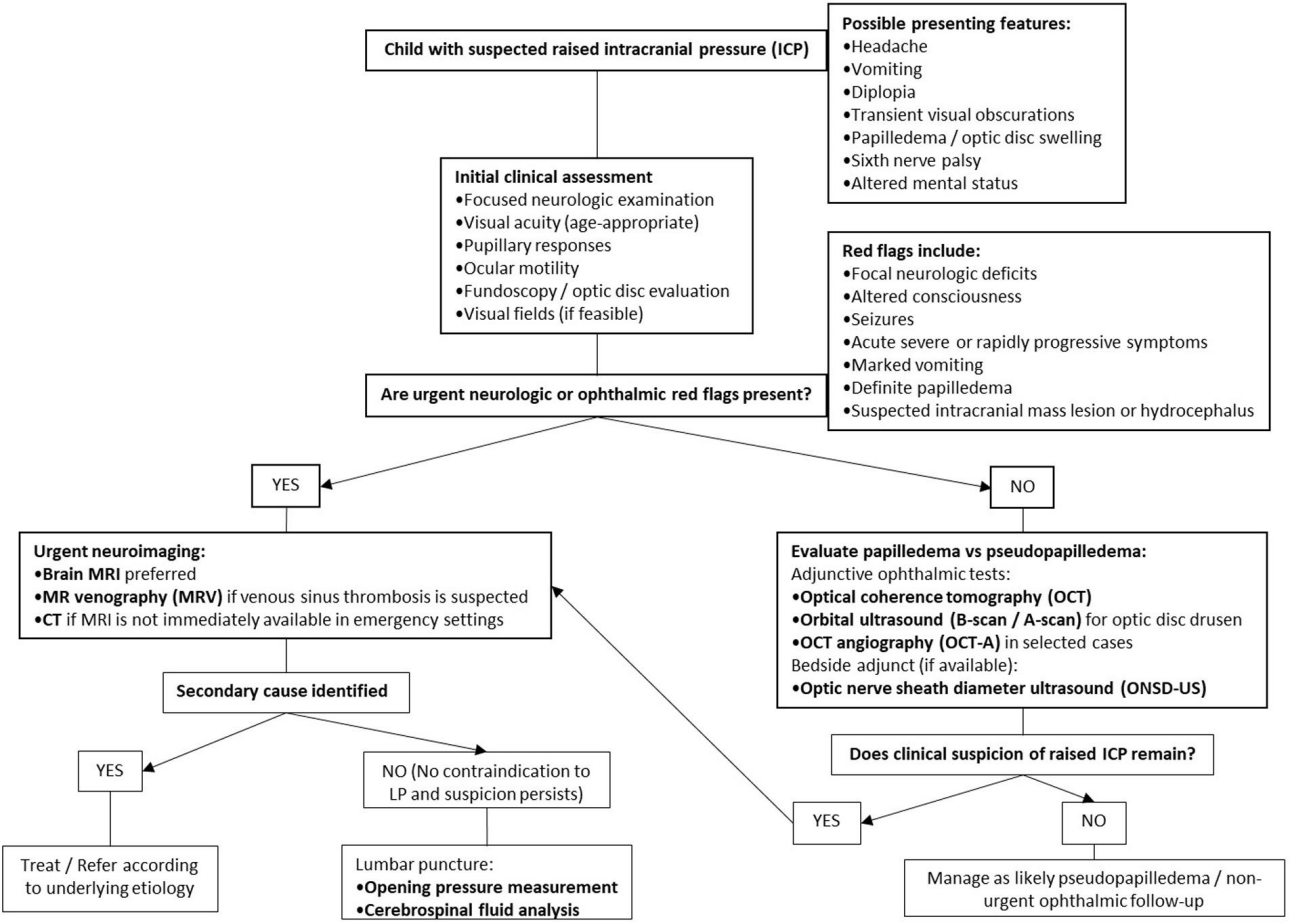
Proposed diagnostic workflow for suspected raised intracranial pressure in children.

A practical stepwise approach to the evaluation of children with suspected raised ICP is required (Figure 3). Initial neurologic and ophthalmic assessment should guide the urgency of neuroimaging, particularly when red flags or definite papilledema are present. In cases of equivocal optic disc swelling, adjunctive ophthalmic modalities such as OCT, orbital ultrasound, and selected OCT-A may help distinguish papilledema from pseudopapilledema. OSND ultrasound may provide rapid bedside support but should be interpreted as an adjunctive rather than definitive test. When neuroimaging excludes contraindications and suspicion persists, lumbar puncture with opening pressure measurement and CSF analysis remains an important confirmatory step. Final diagnosis should rely on integrated clinical, ophthalmic, imaging, and cerebrospinal fluid findings.

## 7 Innovations and future perspectives

As the understanding of increased ICP in children continues to evolve, significant innovations in diagnostic technology offer exciting opportunities to improve both the precision and efficiency of early detection. These advancements in ophthalmologic tools and techniques aim to overcome the inherent challenges associated with pediatric populations.

### 7.1 Automated algorithms for optic nerve sheath diameter measurement

One of the most promising areas in ICP diagnosis is the development of automated algorithms for measuring ONSD, a widely used parameter for assessing raised ICP. Traditionally, measuring ONSD requires ultrasound, which is operator-dependent and can be challenging, particularly in pediatric patients. Variability in measurement results can occur due to factors like the child's cooperation, the skill of the operator, and the positioning of the probe. These inconsistencies in manual ONSD assessment can affect ONSD and the corresponding ONSD cutoff values for the diagnosis of elevated ICP, hereby hampering the full potential of ONSD (151).

To address these limitations, several automated and semi-automated algorithms have been developed in recent years, aiming to improve accuracy, reproducibility, and clinical applicability. Early algorithmic approaches focused on classical image processing techniques applied to B-mode ultrasound images and demonstrated good agreement with expert manual measurements, highlighting the potential of automation to standardize ONSD assessment (151, 152).

More recent developments have incorporated deep learning–based segmentation and video analysis to further enhance performance. Xiao introduced a convolutional neural network–based framework capable of automatically identifying the optic nerve and measuring ONSD from transorbital ultrasound images (153). A review by Escamilla-Ocañas et al. (154) comprehensively evaluated automated ultrasonographic ONSD measurement techniques, emphasizing that although most automated algorithms demonstrate high agreement with expert measurements, external validation, standardization of acquisition protocols, and clinical outcome correlation remain limited.

Automated algorithms for ONSD measurement represent a rapidly evolving field with significant potential to enhance the reliability and clinical utility of ocular ultrasound in increased ICP. Future research should focus on multicenter validation and be integrated into clinical workflows to provide immediate feedback to clinicians, allowing for faster decision-making and intervention.

### 7.2 Automated papilledema detection using deep learning

In recent years, deep learning techniques have been increasingly applied to ophthalmologic imaging, particularly in the automated detection of papilledema. Deep learning algorithms can analyze fundus photographs with remarkable accuracy, identifying subtle changes in the optic disc that may not be readily apparent to human examiners. These algorithms are trained on large datasets of annotated images and can learn to distinguish papilledema from other causes of optic disc swelling, such as optic neuropathy or congenital optic disc anomalies.

One of the first comprehensive automated frameworks was introduced by Echegaray et al., (155) who developed an algorithm for automated analysis of optic nerve head images to detect and stage papilledema using fundus photographs with an accuracy comparable to grading by a neuro-ophthalmologist. Building on this concept, other authors further refined this approach and improved the diagnostic performance, emphasizing the importance of vessel-related features in papilledema detection (156, 157).

An important extension of automated optic disc edema analysis is the ability to distinguish true papilledema from pseudopapilledema. Sathianvichitr et al. (158) developed a deep learning model trained on color fundus photographs to accurately discriminate optic disc drusen from papilledema. Their system achieved high diagnostic performance across multicenter datasets, demonstrating that convolutional neural networks can identify subtle morphological cues on fundus images that are often challenging even for experienced clinicians. This capability is particularly relevant in pediatric populations, in whom optic disc drusen frequently mimic papilledema and may lead to unnecessary neuroimaging or invasive investigations.

Recently, Rambabu et al. (159) conducted a systematic review evaluating AI-based detection of papilledema as a marker of raised ICP. The authors reported consistently high diagnostic performance across studies while highlighting substantial heterogeneity in imaging modalities, reference standards, and validation strategies. Importantly, they noted the relative paucity of pediatric-specific datasets and external validation, limiting immediate generalizability to children with increased ICP.

Most recently, Shapiro et al. (160) explored the application of AI-assisted papilledema diagnosis in rural healthcare settings, demonstrating that automated systems could support clinical decision-making where access to specialist care is limited. Although not focused exclusively on pediatric populations, this work highlights the potential role of automated papilledema detection as a triage and screening tool in underserved environments, which is particularly relevant for children presenting with nonspecific symptoms of raised ICP.

For pediatric patients, automated detection of papilledema holds particular promise, although most existing models have been developed and validated predominantly in adult cohorts. Deep learning models should be designed to account for the normal anatomical differences in the pediatric eye, adjusting for factors such as age-related changes in optic disc size and shape. Furthermore, it can reduce the reliance on highly specialized personnel, thus facilitating timely diagnosis and treatment.

### 7.3 Widefield fundus imaging adapted for pediatric use

Another development in the field of pediatric ophthalmology is the use of widefield fundus imaging, which allows for the capture of detailed images of the entire retina and optic disc in a single, non-invasive scan. Traditional fundus photography provides only a limited view of the retina, which may miss subtle changes or peripheral findings associated with increased ICP. Widefield imaging, however, provides a more comprehensive view, facilitating the detection of additional signs of increased ICP, such as retinal hemorrhages or venous engorgement, which can sometimes be overlooked in standard examinations.

Widefield imaging systems are being adapted for pediatric use, with specialized instruments designed to accommodate the smaller anatomy of children's eyes. These systems are lightweight, easy to use, and capable of capturing high-resolution images quickly, which is crucial when examining uncooperative or young patients. Additionally, the images obtained can be used to monitor changes over time, providing valuable data on the progression of ICP and the response to treatment (161, 162).

## 8 Conclusions

The ophthalmologic assessment remains a vital component in the diagnostic evaluation of children with suspected elevated ICP (Table 5). Through a careful examination of the fundus and optic disc, signs such as papilledema, retinal hemorrhages, and venous engorgement can provide critical clues about the presence of elevated ICP. However, various challenges, including atypical clinical presentations and the variability of normal ocular findings by age, make diagnosis more complex in the pediatric population.

**Table 5:** Take-home messages.

Take-home messages
In children, raised intracranial pressure (ICP) may present with non-specific symptoms. Ophthalmologic assessment can provide early objective clues prompting urgent neuroimaging and/or lumbar puncture when appropriate.
Papilledema is highly suggestive of raised ICP but absence does not exclude elevated ICP, especially in younger children.
Dilated fundus exam and fundus photography, optical coherence tomography (OCT) and optic nerve sheath ultrasound are the most accessible non-invasive tools to support decision-making.
Magnetic resonance imaging is essential to rule out secondary causes (mass lesion, venous sinus thrombosis) and identify supportive signs of increased ICP.
Structural changes may precede functional loss. Hence, serial OCT and age-appropriate visual function testing are key for follow-up.
Pseudopapilledema (disc drusen/crowded discs) is a major confounder in pediatrics; multimodal imaging improves diagnostic certainty and avoids unnecessary invasive work-up.

The unique challenges presented by the pediatric population underscore the need for age-specific considerations when performing ophthalmologic assessments. Children, especially infants and younger children, often exhibit nonspecific or atypical clinical signs of high ICP, which can delay early recognition of this life-threatening condition. In addition, the difficulty of performing an eye examination in uncooperative patients, along with the variability in normal ophthalmologic findings across different age groups, further complicates diagnosis.

Despite these challenges, the correlations between ophthalmologic findings and ICP are well-documented, particularly in the case of papilledema. When detected early, these signs can serve as a key alert for further diagnostic testing, including imaging studies to confirm the presence of increased ICP and assess its underlying causes. Early intervention is crucial, as untreated elevated ICP can result in permanent neurological damage or even death. Thus, the ophthalmologic examination is not only a diagnostic tool but also a critical determinant of clinical outcomes, allowing clinicians to make timely decisions about the need for more invasive diagnostic procedures and therapeutic interventions.

Looking ahead, there are several areas where further research may help improve the accuracy and reliability of ophthalmologic assessments in the diagnosis of ICP in children. Standardizing reference ranges for normal ocular findings across different age groups, as well as exploring non-invasive technologies that can enhance the sensitivity of early increased ICP detection, are important avenues for future investigation. Additionally, further studies should aim to explore the correlation between ophthalmologic findings and ICP levels more precisely, helping to refine the thresholds at which intervention is required.

Ultimately, improving our understanding of the interplay between ophthalmologic findings and ICP in children will lead to better early detection, more accurate diagnoses, and improved clinical outcomes for pediatric patients.
